# Socio-demographic and environmental risk factors associated with multiple under-five child loss among mothers in Bangladesh

**DOI:** 10.1186/s12887-021-03034-y

**Published:** 2021-12-15

**Authors:** Rasel Kabir, Marwa Farag, Hyun Ja Lim, Nigatu Geda, Cindy Feng

**Affiliations:** 1 Collaborative Biostatistics Program, School of Public Health, University of Saskatchewan, Saskatoon, Canada; 2 School of Public Health, University of Saskatchewan, Saskatoon, Canada; 3 School of Public Administration and Development Economics (SPADE), Doha Institute for Graduate Studies, Doha, Qatar; 4 Department of Community Health and Epidemiology, College of Medicine, University of Saskatchewan, Saskatoon, Canada; 5 Center for Population Studies, College of Development Studies, Addis Ababa University, Addis Ababa, Ethiopia; 6 Department of Community Health and Epidemiology, Faculty of Medicine, Dalhousie University, Halifax, Nova Scotia Canada

**Keywords:** Under-five child mortality, Count regression, Complex survey design, Environmental factors

## Abstract

**Background:**

Despite the substantial decline in child mortality globally over the last decade, reducing neonatal and under-five mortality in Bangladesh remains a challenge. Mothers who experienced multiple child losses could have substantial adverse personal and public health consequences. Hence, prevention of child loss would be extremely desirable during women’s reproductive years. The main objective of this study was to determine the risk factors associated with multiple under-five child loss from the same mother in Bangladesh.

**Methods:**

In this study, a total of 15,877 eligible women who had given birth at least once were identified from the 2014 Bangladesh Demographic and Health Survey. A variety of count regression models were considered for identifying socio-demographic and environmental factors associated with multiple child loss measured as the number of lifetime under-five child mortality (U5M) experienced per woman.

**Results:**

Of the total sample, approximately one-fifth (18.9%, *n* = 3003) of mothers experienced at least one child’s death during their reproductive period. The regression analysis results revealed that women in non-Muslim families, with smaller household sizes, with lower education, who were more advanced in their childbearing years, and from an unhygienic environment were at significantly higher risk of experiencing offspring mortality. This study also identified the J-shaped effect of age at first birth on the risk of U5M.

**Conclusions:**

This study documented that low education, poor socio-economic status, extremely young or old age at first birth, and an unhygienic environment significantly contributed to U5M per mother. Therefore, improving women’s educational attainment and socio-economic status, prompting appropriate timing of pregnancy during reproductive life span, and increasing access to healthy sanitation are recommended as possible interventions for reducing under-five child mortality from a mother. Our findings point to the need for health policy decision-makers to target interventions for socio-economically vulnerable women in Bangladesh.

## Background

Ensuring that children survive infancy is an important population health indicator. Child mortality is a great concern in many developing countries. One of the suggested health targets under the Millennium Development Goals (MDGs) was to end preventable newborn deaths by lowering infant mortality to 48 per 1000 live births and under-five mortality (U5M) to 31 per 1000 live births by 2015, in all countries. Under the Sustainable Development Goals (SDGs), the further target is to lower the newborn mortality rate to 12 or fewer per 1000 live births and the U5M to 25 or fewer per 1000 live births by 2030 [1]. Although there has been substantial global progress in reducing U5M, it is still a major public health problem in many developing countries. In Bangladesh, the infant and U5M rates were still relatively high, i.e., 38 per 1000 live births and 46 per live births, respectively (BDHS 2014, NIPORT) [2], so substantial effort is still in great need to achieve the SDGs. This warrants examination of factors associated with U5M in the country to mitigate the risk of mortality and improve child survival.

It is well documented that mortality rates and risk factors vary according to demographic and socio-economic characteristics, such as region [3, 4], places of residence [5], education, wealth index [6], age at first birth [7, 8], and religion [3, 9]. World Health Organization (WHO) refers all women aged 15–49 years as women of reproductive age [10]. However, evidence shows that confining reproduction between 20 and 34 years old [11], and WHO-recommended waiting period of 2–3 years for between pregnancies [12] may significantly decrease both child and maternal mortality. A lack of access to potable water and sanitary facilities for children under 5 years old is a serious public health problem in many developing countries. The public health literature has identified unimproved water and sanitation as leading causes of childhood diarrhea and childhood death. Other studies have shown that access to improved water and sanitation leads to a reduction in both [13–15].

Despite the existing studies on child mortality, to the best of our knowledge, none of the previous studies in Bangladesh examined multiple under-five child loss per mother had during her reproductive years. Most of the studies conducted in Bangladesh have focused on U5M by treating the deaths of children from the same mother as independent observations [4, 16–19]. From a statistical perspective, an intrinsic correlation between the children from the same mother is expected due to the shared biological and genetic factors. However, ignoring the correlations between repeated losses of children can lead to invalid inferences [20, 21]. In addition, this line of research is significant for applied settings because the mothers at risk for the more adverse short and long-term consequences from child loss could be those mothers who had multiple child loss. Moreover, ignoring complex sampling design features, including sampling weight, in the statistical analysis of previous studies might lead to substantial differences in inferences [22, 23]. The main objective of the present study was to determine the risk factors for multiple U5M experienced by women in their child bearing age based on nationally representative data from Bangladesh, accounting for complex survey design to produce more efficient estimates and valid inferences.

## Methods

### Data sources

Data for this study were based on the nationally representative secondary data from the 2014 Bangladesh Demographic and Health Survey (BDHS). The survey is primarily intended to serve as a source of population and health data for policymakers and researchers in Bangladesh. The survey was conducted by Mitra and Associates, a Bangladeshi research firm, under the authority of the National Institute for Population Research and Training. The U.S. Agency for International Development and Inner-City Fund (ICF) International provided financial and technical support, respectively.

### Sample design

The 2014 BDHS sample is nationally representative and covers the entire population, except those residing in public and private institutions (e.g., prisons and mental health institutions). This cross-sectional survey used a sampling frame prepared from the list of enumeration areas (EAs) in Bangladesh’s 2011 Population and Housing Census. The Bangladesh Bureau of Statistics (BBS) provided the list of EAs. An EA with an average of 120 households was the primary sampling unit (PSU) for this survey. Bangladesh has seven administrative divisions: Barisal, Chittagong, Dhaka, Khulna, Rajshahi, Rangpur, and Sylhet.

Two-stage stratified sampling of households was used in this survey. In the first stage, probability proportional to the EA size was used to select 600 EAs, 207 in urban areas and 393 in rural areas. A complete household listing operation was then carried out in all of the selected EAs to provide a sampling frame for the second-stage of household selection. In the second stage of sampling, a systematic sample of 30 households on average was selected per EA to provide statistically reliable estimates of key demographic and health variables for the country, for urban and rural areas separately, and for each of the seven divisions. Overall, 18,000 residential households were selected [2]. This study only included mothers who had a record of children ever born. Individuals with missing values of the variables of interest were excluded from the final analysis, leading to a final sample size of 15,877 mothers.

### Variables

#### Dependent variable

The dependent variable for this study is the total number of children who died before reaching the age of five per mother among the mothers who had given birth to at least one child during their reproductive years. The difference between the number of children ever born and the number of living children was used to calculate the number of children lost per mother. The total number of children ever born was included in the count regression model as the offset term.

#### Independent variables

The independent variables were broadly classified as individual, household, community-level factors, and environmental risk factors. The individual-level variables under consideration were the respondent’s education level recoded as no education, primary and above primary; Body Mass Index (BMI) with three-level categories: normal, underweight, overweight; age at first marriage measured in years; age at first birth measured in years; and time interval from marriage to interview date calculated as years. Household-level factors included the sex of the head of household (male versus female); wealth index recoded as poor, middle class and rich; religion (Muslim versus non-Muslim); household size categorized into three groups:1–3, 4–6 and 7+, and media exposure (yes versus no). Community-level factors included the place of residence (rural versus urban) and division (determined by individual’s participating division from one of the seven divisions: Barisal, Chittagong, Dhaka, Khulna, Rajshahi, Rangpur, and Sylhet). The survey also included environmental factors known to increase the risk of illness and death among children, including having clean water and improved toilet facilities. The sources of drinking water and toilet facility were recoded as two-level variables: improved versus non-improved.

### Statistical analyses

Count regression models were employed for modeling the number of U5M per mother, including Poisson, negative binomial (NB), zero-inflated Poisson [24] and zero-inflated NB [25] models with the number of live births per woman during their childbearing years as the offset term. Zero-inflated models provide a way of modeling the excessive proportion of zero values by allowing overdispersion. AIC was used to identify the appropriate count model among the considered count models.

To screen the covariates, we examined the univariable association between each risk factor and the outcome. Variables where the *p*-value was < 0.2 are retained in the initial multivariable model to avoid excluding important variables from the model. The manual backward selection was used to develop the main effects model, retaining only variables where *p* < 0.05. In the final step, all the covariates remaining in the final model were tested for significant interactions. After selecting the covariates, the models were compared using the Akaike Information Criterion (AIC) for selecting the best model. The association between main effects and the outcome in the final model were reported as adjusted relative risk (RR) with 95% confidence intervals (95% CI) and *p*-values. The analyses were carried out using *svyglm* function from the survey package in the R Statistical Software (Foundation for Statistical Computing, Vienna, Austria). The complex survey design was incorporated in the analysis by using the *svydesign* function from the survey package.

## Results

### Descriptive statistics

The distribution of the number of U5M per mother is presented in Table 1, which shows that about 81.1% of the mothers had no U5M in their lifetime, while 3003 of 15,877 (18.9%) women reported the loss of at least one child. The distribution is highly right-skewed, with 124 (0.78%) mothers reported deaths of 3 children and 44 (0.28%) mothers reported lost at least four children.

**Table 1:** Distribution of the number of under-five child loss (U5M) per mother in Bangladesh, 2014, *n* = 15,877

Number of U5M per mother	Number of mothers	Percentage
0	12,874	81.09
1	2300	14.49
2	535	3.37
3	124	0.78
4+	44	0.28

Table 2 presents the sample characteristics for the entire sample and for those respondents who experienced at least one child death, respectively. Respondent’s age (mean ± sd) was 32.11 ± 8.81, while the average age at first marriage was 15.66, which is lower than the legal age of 18 years. Respondents’ median age at first birth was 17 years old with an interquartile range (IQR) of 3. Only 44.54% of the women surveyed had secondary education or higher, while 25.2% had no education. Of these, 32.5% had lost at least one child in their lifetime. For mothers with secondary education and higher, less than one-tenth (9.83%) had lost a child. This pattern repeats itself for the education level of their partners, where U5M was higher for mothers whose husbands had little or no education. More respondents lived in rural areas (65.94%) than in urban areas (34.06%). About 21% of women living in rural areas experienced the death of at least one child during their childbearing period, which was higher than urban areas (14.90%). 13.52% of mothers from wealthy households have reported a loss of at least one child. In contrast, 24.42% of mothers who belonged to the poorest household reported having lost at least one child during their reproductive years. The survey also showed that mothers exposed to media had lower rates of child mortality (15.13%) compared to mothers (25.14%) who did not have access to any form of media (radio, television or newspaper). More than half (56.82%) of the mothers surveyed had a normal BMI and the percentages for underweight and overweight mothers were 17.93 and 25.25%, respectively. However, the U5M rate among underweight mothers was higher (22.30%) than the other two categories. In terms of geographical variations in child mortality across divisions, most divisions had a similar rate, ranging from 16.29 to 19.22%, while 25.59% of mothers in Sylhet had experienced U5M. Mothers without access of improved toilet facilities accounted for 19.70% of U5M, while mothers with improved toilet facilities accounted for 18.26% of U5M. The rate of child mortality was higher (19.82%) in families with access to an improved source of drinking water than those with access to a non-improved source of water (14.26%).

**Table 2:** Sample characteristics for variables included in the analysis and for those respondents who experienced at least one under-five child loss, *n* = 15,877

Variables name	n (%)	U5M ≥ 1(%)	*P*-value*	*P*-value
***Individual-level risk factors***				
**Respondent’s education level**				
No education	4009 (25.25)	32.5		
Primary	4797 (30.21)	20.95	< 0.001	< 0.001
Secondary/higher	7071 (44.54)	9.83		
**Partner’s education level**				
No education	4747 (29.90)	27.58		< 0.001
Primary	4407 (27.76)	20.04	< 0.001	
Secondary/higher	6723 (42.34)	12.06		
**Respondent’s Body Mass Index (BMI)**				
Normal	9022 (56.82)	19.29		< 0.001
Underweight	2847 (17.93)	22.3	< 0.001	
Overweight	3266 (25.25)	15.67		
**Age at first marriage (median in years, IQR**^**a**^**)**	15, 3	15, 3	< 0.001	< 0.001
**Age at first birth (median in years, IQR**^**a**^**)**	17, 3	17, 4	< 0.001	< 0.001
**Marriage to interview date (median in years, IQR**^**a**^**)**	15.5, 15	23, 13.58	< 0.001	< 0.001
***Household-level risk factors***				
**Household head**				
Male	14,033 (88.39)	18.96	0.739	0.105
Female	1844 (11.61))	18.6		
**Household size**				
1–3	3735 (23.52)	18.26		
4–6	9144 (57.59)	18.89	0.269	< 0.001
7+	2998 (18.88)	19.81		
**Religion**				
Non -muslim	1557 (9.81)	18.43	0.634	0.009
Muslim	14,320 (90.19)	18.97		
**Wealth Index**				
Rich	6679 (42.07)	13.52		
Middle class	3208 (20.21)	19.86	< 0.001	< 0.001
Poor	5990 (37.72)	24.42		
**Media exposure**				
Yes	9875 (62.20)	15.13	< 0.001	< 0.001
No	6002 (37.80)	25.14		
***Community-level risk factors***				
**Residential Place**				
Urban	5408 (34.06)	14.9	< 0.001	0.011
Rural	10,469 (65.94)	20.99		
**Division**				
Barisal	1889 (11.90)	19.22		
Chittagong	2532 (15.95)	19.04		
Dhaka	2734 (17.22)	17.3	< 0.001	0.014
Khulna	2314 (14.57)	16.29		
Rajshahi	2260 (14.23)	18.63		
Rangpur	2280 (14.36)	17.94		
Sylhet	1868 (11.77)	25.59		
***Environmental risk factors***				
**Sources of drinking water**				
Improved source of water	13,320 (83.70)	19.82	< 0.001	0.045
Non-improved source of water	2557 (17.30)	14.26		
**Toilet facilities**				
Improved toilet facilities	8662 (54.56)	18.26	0.023	0.009
Non-improved toilet facilities	7215 (45.44)	19.7		

*P-value was calculated from t-test for continuous variables and chi-square test for categorical variable according to the groups of mothers experiencing at least one child loss versus mothers with no child loss ^a^ IQR indicates interquartile range; *P*-value in the last column was from univariable Poisson model

### Multivariable analysis

**Fig. 1:**
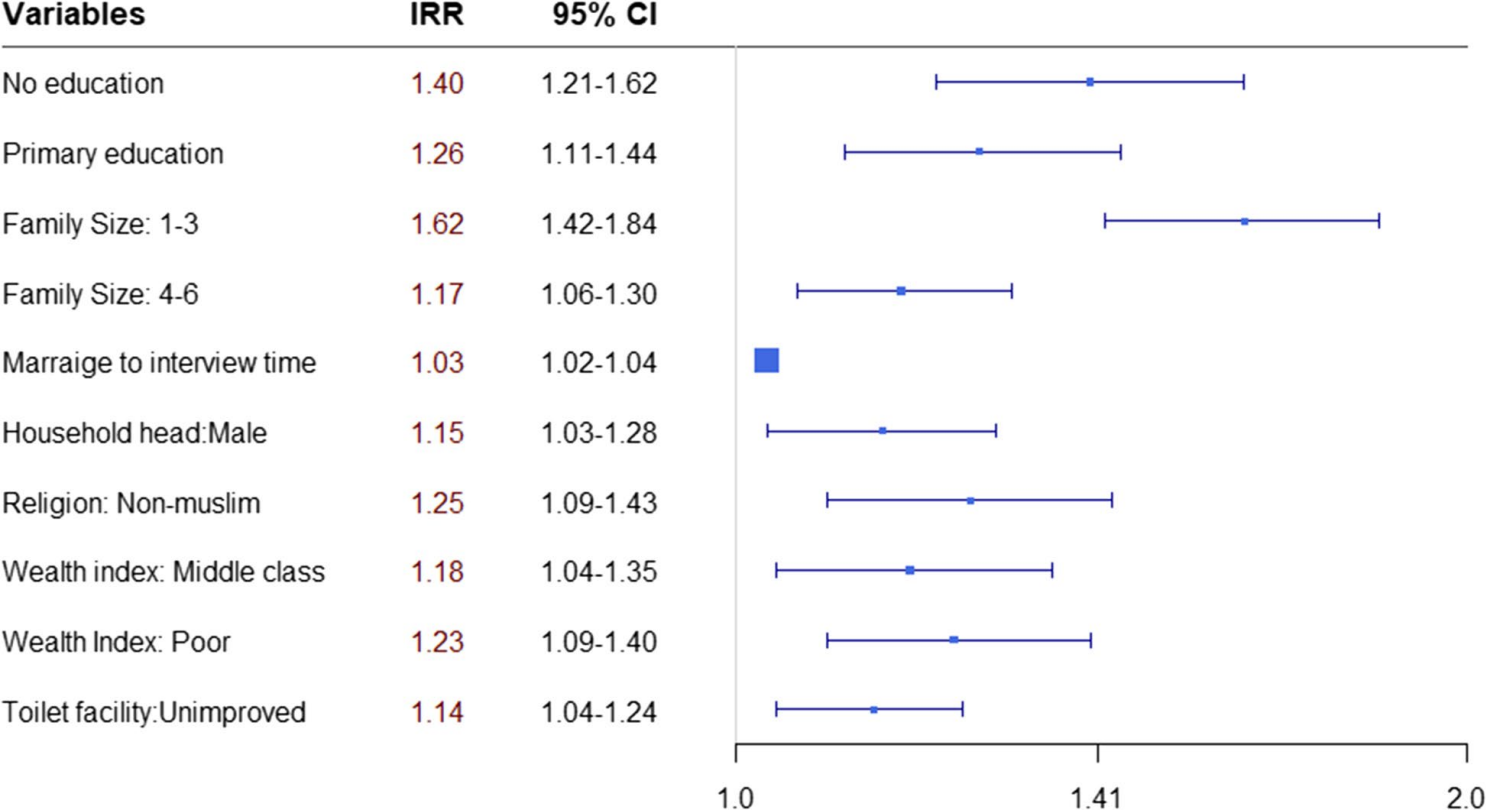
Plot of Incidence Rate Ratio (IRR) based on the multivariable Poisson regression model

**Fig. 2:**
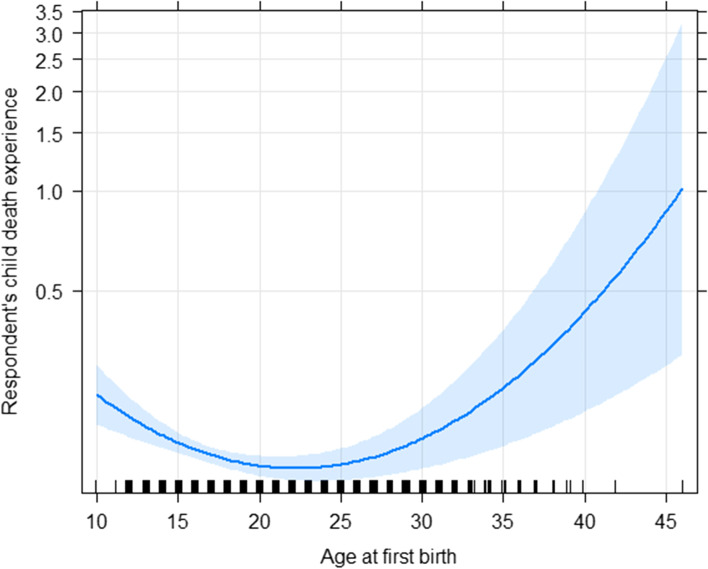
Nonlinear effect of age at first birth on the risk of child death based on the multivariable Poisson regression model

Count regression models, including Poisson, NB, and their zero-inflated counterpart models, were used for modeling the number of child deaths a mother reported during her childbearing years. The AIC values for all the considered models are almost identical, which implies that the data are not zero-inflated or overdispersed relative to the Poisson model. Therefore, the Poisson model was selected as the best-fitting model. We also examined the nonlinearity effects of the continuous variables, i.e., age at first birth and time from first marriage to interview date, by modeling their effects as spline functions. The backward elimination approach was used to select the variables in the final model, which included a respondent’s education level, household size, natural spline of age, time from marriage to interview date, religion, sex of household head, and wealth index (Table 3). We also checked the pairwise interaction terms, but none was found significant. The covariate effects are also depicted in Fig. 1 for ease of comparison. The nonlinear effect of age at first birth is presented in Fig. 2.

**Table 3:** Multivariable Poisson regression for under-5 child mortality

Variables name		95% CI of IRR		
	IRR	Lower	Upper	*P*-value
***Individual-level risk factors***				
Respondent’s Education Level				
Secondary/higher*				
Primary	1.26	1.11	1.44	< 0.001
No education	1.40	1.21	1.62	< 0.001
Household Size				
7 + *				
4–6	1.17	1.06	1.30	0.003
1–3	1.62	1.42	1.84	< 0.001
Marriage time to interview date (reproductive time in year)	1.03	1.03	1.04	< 0.001
***Household-level risk factors***				
Household head				
Female^a^				
Male	1.15	1.03	1.28	0.013
Religion				
Muslim^a^				
Non-muslim	1.25	1.09	1.43	0.002
Wealth Index				
Rich^a^				
Middle class	1.18	1.04	1.35	0.012
Poor	1.23	1.09	1.40	0.001
***Environmental risk factors***				
Toilet facilities				
Improved facilities^a^				
Non-improved facilities	1.14	1.04	1.24	0.003

^a^Reference group

The incidence rate ratio (IRR) reported in the third column of Table 3 revealed that mothers who had no education experienced child loss 40% (IRR = 1.40, 95% CI:1.21–1.62) higher than mothers with secondary school or higher. Primary education was associated with a 26% (IRR = 1.26, 95% CI:1.11–1.44) increased risk of child death compared to mothers with more than primary education, keeping all other covariates constant. The risk of U5M increased as household size decreased. The adjusted risk of mortality for a child born in a household of 1–3 individuals and 4–6 was 1.62 (IRR = 1.62, 95% CI: 1.42–1.84) and 1.17 (IRR = 1.17, 95% CI: 1.06–1.30) times higher, respectively, relative to those born in a household with seven or more members.

The results indicate a linear effect of time from first marriage to interview date and a strong non-linear effect of age at first birth. Specifically, the results revealed that mothers for whom more years had passed from marriage to the survey date had lived for more potential childbearing years, which increased the likelihood of having experienced child mortality. The risk of child death per mother increased by 3% for each year increase between marriage and the survey date.

A “J-shaped” trend demonstrated in Fig. 2 displayed the effect of age at first birth on the risk of U5M. The figure indicates that the risk of U5M trends tends to be higher for earlier mother’s age at first birth followed by a decreasing trend up to about 25 years old but increases again for increasing mother’s age at first birth after 25 years old.

Mothers who had less access to basic sanitary facilities suffered an increase in child death in their childbearing years. Table 3 shows the rate of U5M is 14% higher [IRR = 1.14, CI:1.04–1.24, *p*-value = 0.003] for mothers using poor toilet facilities than mothers with access to improved sanitary facilities. The analysis found a significant relationship between religion and child loss. The risk of child mortality among the Muslim mother was 20% lower compared to non-Muslim women (IRR = 0.80, CI:0.70–0.92, *p*-value = 0.002). Regional variation, age at first marriage, partner’s educational level, and respondent’s BMI were not associated with child mortality.

## Discussion

This is the first study in Bangladesh to determine risk factors associated with the number of child loss per mother based on a large and nationally representative database. Previous studies on U5M in Bangladesh and elsewhere often model the current U5M of mothers as a binary outcome [4, 16–19]. This tends to ignore the clustering nature of early mortality in a single individual mother. Mothers’ multiple experiences of stillbirth, infant death, or child death will often lead to exacerbated short-term postnatal depression, tension, and anxiety [26]. It could also have long-term mental/physical health repercussions (such as insomnia, somatic symptoms, suicidal inclinations, social dysfunction, and secondary social consequences) due to increased social pressures [27]. Hence, the total number of child loss experienced by a mother could reflect the severity of the problem, providing a unique contribution for designing targeted interventions to the mothers who are highly vulnerable experiencing child loss during their reproductive ages.

Our study affirmed the women who had no education or primary education were more likely to experience a higher U5M rate than those who had a higher level of education. Previous studies indicated that education improves the ability of mothers to implement basic health knowledge, such as nutrition [28], and immunization [29] and that education facilitates their capacity to navigate through their environment, including using health care facilities, effectively communicating with health professionals, complying with treatment recommendations and keeping their environment clean. Furthermore, women who are comparatively well educated often have greater control over health choices for their children [30].

Household socio-economic status is also significantly associated with U5M rates per mother [31, 32]. Our results indicate that children of mothers who were from lower-income households have a significantly higher risk of mortality than children of mothers from higher wealth index households. The risk of child mortality also increases as household size decreases. Women living in small households experience greater child loss than in larger households. This result is in line with a study conducted by Berhie [33] and Zoya [34] but inconsistent with the results from Ethiopia that showed a negative relationship between household size and child mortality [35].

The present study also documented a weak association between household headship and child death. Mothers from female-headed households were reported to have less experience with child loss compared to mothers from male-headed households. This finding is consistent with Gupta et al.’s study [36]. Islam was associated with lower U5M rates compared to other faiths. Increased U5M could be a result of certain practices and superstitions associated with non-Muslim religions [37]. This finding may also be accounted for the differential social and economic situations of the religious groups in Bangladesh. Muslim group probably coupling with mainly middle and upper class educated partners might have access to improved medical and health care facilities, and their wider childcare knowledge may also contribute to this differential finding. Our findings are consistent with Brainerd et al.’s study, which suggests that Muslim infants have an advantage over Hindu infants in height-for-age [38]. An early Bangladeshi study also reported lower child mortality among Muslim women than non-muslim [39]. Nevertheless, the sample was much skewed with only 10% of respondents being non-Muslim. Other unobserved factors may influence this relationship. As a result, futher studies are warranted.

It is evidenced from the analysis that the age at first birth was significantly associated with U5M mortality rate. Our result was consistent with Finlay et al.’s study that reported that adolescent mothers’ first children are vulnerable to child death and poor health outcomes [7, 40–43]. The previous study also showed that older mother (aged 30 or older) were at greater risk of experiencing under-five child mortality compared to mothers whose age at the time compared to mothers age of 24 or less [43]. In this present study, a non-linear association was detected. The U5M mortality rate decreased as age at first birth increased up to about 25 years of old, followed by a drastic increasing trend as age at first birth increases. This finding adds to the existing literature by giving a more accurate estimate of the nonlinear effect of age at first birth.

Interestingly, the type of toilet facilities a woman had access to was a statistically significant factor for U5M rate per mother. Women living in households that used composting toilets, pit latrine types of toilets, or no toilets experienced higher under-5 mortality than those who lived in households with flush or otherwise modern toilet facilities. A recent study based on evidence from subnational panel data in 59 countries predicted large reductions in diarrhea prevalence and child mortality due to improvements in sanitation. These studies estimated that sanitation improvements could account for just under 10% decline in child mortality from 1990 to 2015 [44]. Although there is a significant improvement over the last two decades, the number of open defecators and unhygienic sanitation is still highest in the south Asian region [45]. Thus, targeted interventions to address these factors may contribute to accelerating the reduction of child mortality in Bangladesh.

U5M is a still pressing public health concern in Bangladesh, and this study tried to figure out the critical determinants of U5M experience among Bangladeshi mothers. Programs should promote the empowerment of women through education and broader health training coverage. Policies and interventions aimed at improving maternal education levels could be beneficial to reduce child mortality if assisted with additional supports to improve the living standard and socio-economic condition. Strategies targeted in designing the counselling program to determine an ideal time for a first pregnancy and subsequent birth intervals may further impact the child’s survival. In order to promote a hygienic environment, government, non-government organizations, and other relevant stakeholders should work together to educate mothers about the norms of sanitation, and additional efforts to improve sanitation should be prioritized.

## Conclusion

In summary, our study showed that a significant proportion of mothers experienced under-5 mortality during their reproductive period. The rate of mortality is determined by a range of individual, household and community-level variables. Our study confirms that low education, poor socio-economic condition, extremely young or old age at first birth, and an unhygienic environment are independently associated with U5M experienced by mothers. Bangladesh government should pay more attention to maternal education, including health education, increasing access to healthy sanitation, and promoting appropriate timing of pregnancy during reproductive life span. Our study will also assist health policy decision-makers in planning intervention programs targeting socio-economically vulnerable women in Bangladesh and other developing countries.

### Limitations

While this study had a relatively large sample size, the study also has some limitations, including the cross-sectional nature of the survey. This limits potential inferences about causal relationships between U5M and some of the risk factors examined. The responses from mothers might have also be subjected to recall biases. The information for potential predictors for U5M rate, such as respondent’s anemia status, disease history, family planning, are not available. Due to the time-varying nature of the outcome variable, it was not possible to include some of the health intervention level variables (such as the antenatal visit of the mother, child vaccination, policy to encourage institutional birth, any nutritional program to provide nutrition to pregnant mothers and children, etc.). This was because DHS surveys collect health intervention level variables only for the most recent birth (i.e., births/deaths occurring during the last 3 years prior to the survey). Future efforts should consider collection of longitudinal and population-based cohort study to characterize the profile of each mother and also help use to deeply understand the underlying mechanism for multiple child loss per mother. These limitations need to be taken into consideration when generalizing our results.

## Data Availability

The datasets used for this study are made available from ICF international/DHS program at https://dhsprogram.com/data/Access-Instructions.cfm. Thus, administrative permissions were required to access the raw data from this organization. Public access to the database is open upon permission.

## Abbreviations

AIC: Akaike information criteria
BBS: Bangladesh Bureau of Statistics
BMI: Body Mass Index
EA: Enumeration areas
IRR: Incidence rate ratio
MDGs: Millennium Development Goals
NB: Negative binomial
RR: Risk ratio
SDGs: Sustainable Development Goals
U5M: Under five mortality
